# Mapping the Relative Biological Effectiveness of Proton, Helium and Carbon Ions with High-Throughput Techniques

**DOI:** 10.3390/cancers12123658

**Published:** 2020-12-05

**Authors:** Lawrence Bronk, Fada Guan, Darshana Patel, Duo Ma, Benjamin Kroger, Xiaochun Wang, Kevin Tran, Joycelyn Yiu, Clifford Stephan, Jürgen Debus, Amir Abdollahi, Oliver Jäkel, Radhe Mohan, Uwe Titt, David R. Grosshans

**Affiliations:** 1 Department of Experimental Radiation Oncology, The University of Texas MD Anderson Cancer Center, Houston, TX 77030, USA; LBronk@mdanderson.org (L.B.); Benjamin.Kroger@UTSouthwestern.edu (B.K.); kettran@UTMB.EDU (K.T.); jcy4@rice.edu (J.Y.); 2 Department of Radiation Oncology, The University of Texas MD Anderson Cancer Center, Houston, TX 77030, USA; 3 Department of Radiation Physics, The University of Texas MD Anderson Cancer Center, Houston, TX 77030, USA; fguan@mdanderson.org (F.G.); darshana.c.p@gmail.com (D.P.); duo.ma@utsouthwestern.edu (D.M.); xiaochunw@mdanderson.org (X.W.); rmohan@mdanderson.org (R.M.); 4 Texas A&M Institute of Biosciences and Technology High Throughput Research and Screening Center, Houston, TX 77030, USA; cstephan@tamu.edu; 5 National Center for Tumor Diseases, Deutsches Krebsforschungszentrum, 69120 Heidelberg, Germany; juergen.debus@med.uni-heidelberg.de (J.D.); a.amir@dkfz.de (A.A.); o.jaekel@dkfz-heidelberg.de (O.J.); 6 Heidelberger Ionenstrahl Therapiezentrum, Deutsches Krebsforschungszentrum, 69120 Heidelberg, Germany

**Keywords:** relative biological effectiveness, charged particle therapy, high-throughput techniques, lung cancer cells

## Abstract

**Simple Summary:**

Although particle therapy using protons and heavier ions has many inherent advantages when compared to x-rays for cancer treatment, numerous unknowns still exist in the radiobiology of particle therapy. Informative high-accuracy biological effects data are lacking and difficult to obtain. This study aimed to provide a novel high-throughput experimental method to more efficiently obtain large amounts of biophysical data of particle therapy and to correlate the biological responses with the physical characteristics of particle beams.

**Abstract:**

Large amounts of high quality biophysical data are needed to improve current biological effects models but such data are lacking and difficult to obtain. The present study aimed to more efficiently measure the spatial distribution of relative biological effectiveness (RBE) of charged particle beams using a novel high-accuracy and high-throughput experimental platform. Clonogenic survival was selected as the biological endpoint for two lung cancer cell lines, H460 and H1437, irradiated with protons, carbon, and helium ions. Ion-specific multi-step microplate holders were fabricated such that each column of a 96-well microplate is spatially situated at a different location along a particle beam path. Dose, dose-averaged linear energy transfer (LET_d_), and dose-mean lineal energy (y_d_) were calculated using an experimentally validated Geant4-based Monte Carlo system. Cells were irradiated at the Heidelberg Ion Beam Therapy Center (HIT). The experimental results showed that the clonogenic survival curves of all tested ions were y_d_-dependent. Both helium and carbon ions achieved maximum RBEs within specific y_d_ ranges before biological efficacy declined, indicating an overkill effect. For protons, no overkill was observed, but RBE increased distal to the Bragg peak. Measured RBE profiles strongly depend on the physical characteristics such as y_d_ and are ion specific.

## 1. Introduction

In recent years, interest in using heavier charged particles, i.e., protons and carbon ions, in cancer treatment has increased markedly. Globally, charged particle or ion therapy is becoming more commonly used, with over 113 particle therapy facilities currently in clinical operation and many more either under construction or being planned [1,2]. Although the most common form of charged particle therapy at present is with protons, 14 centers in the world are currently using carbon ions and there is also interest in using other ions clinically or even hybrid-ion therapy [3].

The clinical benefits of particle therapy remain controversial owing to associated high costs and the still-unanswered question of realizable therapeutic advantage versus standard photon-based therapies [4,5,6]. However, a growing body of evidence suggests that certain ions present optimal peak-to-entrance-dose biological effects [7,8]. Harnessing the differential biological effects of a given therapeutic ion beam by placing the regions with low biological effect in normal tissues and those with increased efficacy in the tumor volume are the core tenets driving biologically optimized particle therapy. This requires understanding the complex spatial distribution of biological effects of ions, i.e., as a function of particle type and energy, beam characteristics, dose, tissue or cell type, and biological endpoint [9,10,11,12,13]. However, such spatial mapping remains limited because of the low efficiency of traditional experimental techniques.

The initial rationale for using charged particle radiation was based on the lower entrance dose and the finite range of a charged particle beam, which results in improved dose distributions in the target volume while reducing off-target exposures. Moreover, ions heavier than protons are known to exhibit higher relative biological effectiveness (RBE) to reference photons, with the biological response being related to the particle type as well as its energy. As such, for therapy with heavy ions such as carbon, the clinically used RBEs currently are spatially variant and on the order of 2–4, whereas proton therapy practice employs a spatially uniform RBE value of 1.1 and the use of this invariant proton RBE for clinical application is unlikely to be changed in the near future [14,15,16]. However, the RBE values for both protons and carbon continue to be debated. For instance, there are significant differences between reported measured proton RBE values and those predicted by available biological effect models [9,11]. Similarly, although a variable RBE of carbon ions has been used clinically to deploy biological dose optimized treatment plans, different models, i.e., the local effect model (LEM) used in Europe and the modified microdosimetric kinetic model (mMKM) applied in Japan, could generate different RBE values even with the identical setup in making treatment plans [17,18]. An additional challenge is the usage of multiple summary statistics for the condensation of the particle energies present within a volume, such as linear energy transfer (LET) and lineal energy (y), within the literature [19,20]. These issues are major obstacles to the implementation of variable RBE treatment planning, especially for proton therapy [10,11,18,21]. Furthermore, these discrepancies can result in large differences in potential dose profiles for the same target site and further complicate comparisons in the clinical outcomes among different carbon ion or even proton centers [22,23,24,25].

To better understand how the physical characteristics affect the biological responses in particle therapy, our group has developed a high-throughput method for more rapidly generating biological data at a series of locations along a beam path to sample differential biological effects [26,27,28]. Previous work with protons established and validated this approach and demonstrated a unique RBE vs. dose-averaged linear energy transfer (LET_d_) relationship where RBE was found to rapidly rise in a non-linear fashion beyond the Bragg peak. The present study aimed to test the feasibility of our high-throughput strategy in ions heavier than protons, and, in addition, to further validate the reproducibility of the approach at a new facility by using 6 MV photon and proton irradiation of cells obtained from different suppliers. Given the increased interest in and potential benefits of heavy ion particle therapy, the high-accuracy and high-throughput biophysical system developed by our team has the potential to be widely applied by other investigators in the particle therapy radiobiology community, and the methodology is anticipated to provide new insights into the standardization of the experimental methods in studying ion-specific biological effects.

## 2. Results

**Figure 1:**
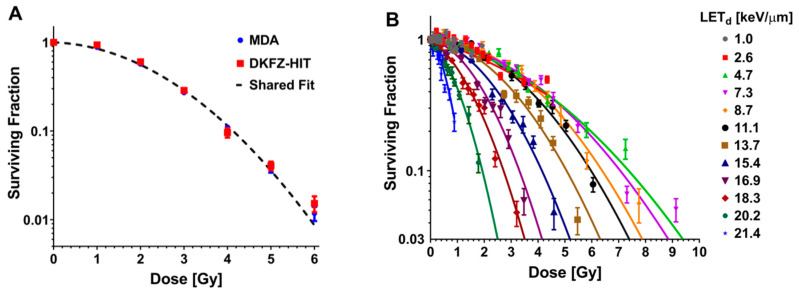

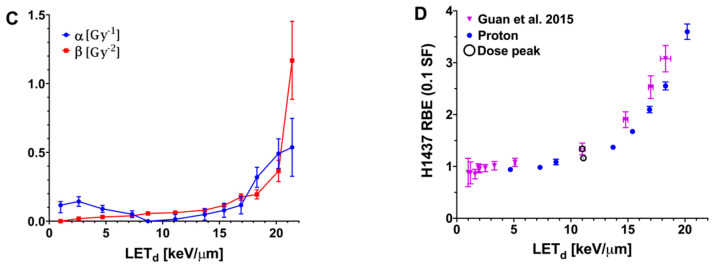
(**A**) The clonogenic survival of two lines of H460 non-small cell lung cancer cells exposed to 6 MV photons was found not to be statistically different between cells obtained from LGC Standards GmbH (Wesel, Germany) and irradiated at Deutsches Krebsforschungszentrum (DKFZ; green), and H460 cells purchased from the American Type Culture Collection (Manassas, VA, USA) and exposed at MD Anderson Cancer Center (MDA; red). *p* < 0.0001; Extra sum-of-squares *F* test. (**B**) High-throughput clonogenic assay results from proton (80.04 MeV) irradiations performed at the Heidelberg Ion Therapy Center for the H1437 non-small cell lung cancer cell line. The H1437 surviving fraction was found to decrease with increasing proton linear energy transfer (LET). Error bars are standard error of the mean. (**C**) α (blue) and β (red) values calculated by fitting the H1437 proton survival data to the linear-quadratic model. (**D**) Relative biological effectiveness (RBE) versus LET_d_ for H1437 cells exposed to protons (blue). RBEs were calculated at the surviving fraction of 0.1 (RBE (0.1 surviving fraction: SF)). Previously published H1437 data are shown for comparison (purple, 79.7 MeV protons). Error bars represent standard error.

To benchmark the response of cells at the two institutions (Heidelberg Ion-Beam Therapy Center (HIT) and MD Anderson), non-small cell lung cancer (NSCLC) H460 cells were irradiated with 6 MV photons. When the high-throughput clonogenic method was used, clonogenic survival was found to be in good agreement between the two institutions, with no statistically significant difference found in the results obtained as determined by the extra sum-of-squares *F* test (Figure 1A). The physical quantity LET_d_ has been utilized in our previous proton irradiation experiments [26]. Our previous work demonstrated agreement of the LET_d_-dependent proton RBE trend of H460 cells measured using the high-throughput clonogenic assay between the two institutions [27]. Therefore, the current proton experiments are presented with associated LET_d_. Using the same physical setup, proton irradiation of NSCLC H1437 cells revealed decreases in surviving fraction (SF) with increasing LET_d_. The SFs were further found to decrease with increasing dose for all LET_d_ exposures sampled (Figure 1B). The lethal α- and sub-lethal β- components from the linear quadratic (LQ) model fit trended similarly increasing with LET_d_ (Figure 1C; Table S1). Our previously generated Cs-137 photon datasets [26] were used to calculate RBE for the present study. The RBEs at an SF of 0.1 (RBE (0.1 SF)) were found to increase throughout the assayed LET_d_ conditions, with a maximum RBE (0.1 SF) of 3.60 and at 20.2 keV/μm (Figure 1D) [26]. Proton entrance RBEs were found to be approximately 1.0 (Supplementary Table S1).

**Figure 2:**
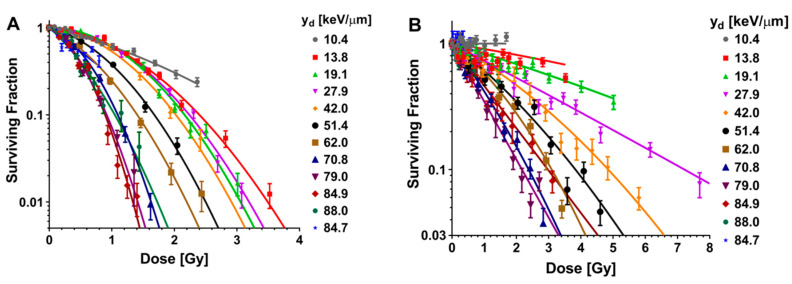

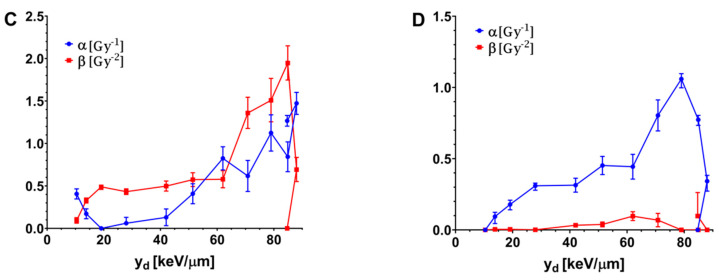
Clonogenic survival results for two non-small cell lung cancer cell lines exposed to helium ions. (**A**) The H460 and (**B**) H1437 cell line results from helium ion irradiations performed at the Heidelberg Ion Therapy Center. A high-throughput irradiation jig was developed to sample locations along a 84 MeV/u helium ion beam. For both cell lines, clonality was found to be increasingly reduced with increasing helium ion y_d_ until 84.9 keV/μm for the H460 line and 79.0 keV/μm for the H1437 line. For exposures to y_d_s higher than these respective values, the surviving fractions were found to begin to increase, indicating a reduced biological effect. Error bars are SEM. (**C**) α (blue) and β (red) values calculated by fitting the H460 clonogenic survival data for cells exposed to helium ions to the linear-quadratic model. (**D**) Linear-quadratic model α (blue) and β (red) values for the H1437 cells exposed to helium ions.

For helium and carbon ion irradiations, the microdosimetric quantity y_d_ was used instead of LET_d_. Irradiation with helium ions led to reductions in SF with the increase in y_d_ until a y_d_ of 84.9 keV/μm for the H460 cell line and 79.0 keV/μm for the H1437 cell line. The SFs then began to increase for beam qualities tested with y_d_ (Figure 2A,B). The LQ model was fit to the clonogenic survival data (Tables S2 and S3). For the H460 cells, α decreased until 19.1 keV/μm, after which it continually increased whereas β increased through 84.9 keV/μm after which it began to decrease (Figure 2C; Table S2). For H1437 cells, irradiation with helium ions led to a continuous increase in α until a y_d_ of 79.0 keV/μm, after which α steadily decreased. The H1437 β-component of irradiation with helium ions was found to be consistently smaller than the α-component (Figure 2D; Table S3).

**Figure 3:**
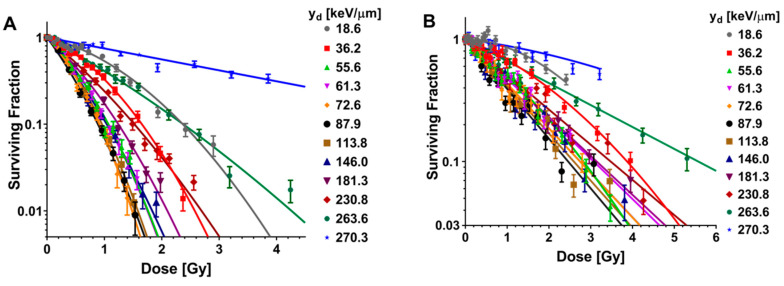

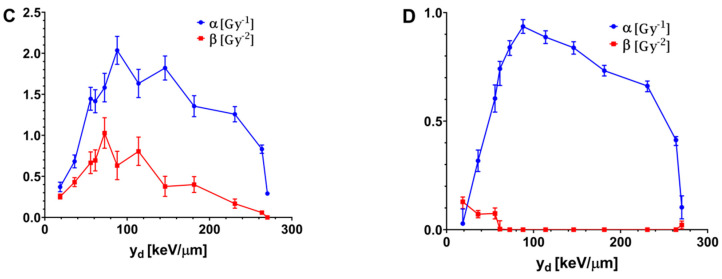
Clonogenic assay results from carbon ion irradiations performed at Heidelberg Ion Therapy Center in the (**A**) H460 and (**B**) H1437 cell lines. A high-throughput irradiation apparatus was designed to simultaneously expose cells to 12 locations along a 153.66 MeV/u carbon ion beam. Similar to the helium ion results (Figure 2), an inflection point was found for carbon ion irradiations at 87.9 keV/μm. The rate of surviving fraction reduction for cells exposed to carbon ions below this LET_d_ was found to continually increase. The rate of surviving fraction reduction began to decrease for exposure to carbon ion y_d_s above 87.9 keV/μm. Error bars are SEM. (**C**) α (blue) and β (red) values calculated by fitting the H460 clonogenic survival data for cells exposed to carbon ions to the linear-quadratic model. (**D**) Linear-quadratic model α (blue) and β (red) values for the H1437 cells exposed to carbon ions.

High-throughput measurements of clonogenic survival were also obtained after irradiation with carbon ions, and data were fit to the LQ model (Figure 3A,B; Tables S4 and S5). The response of both H460 and H1437 cells was found to increase initially and saturate approaching column 6 corresponding to y_d_ value of 87.9 keV/μm and then decrease. The LQ model fits for both cell lines demonstrated similar trends for α (Figure 3C,D). For both cell lines, the lethal α-component increased until 87.9 keV/μm, after which it slowly declined with increasing y_d_. The sub-lethal β-component derived from the LQ model fits for carbon irradiation exhibited different trends between the two cell lines. For the H460 cells, the β-component increased until a y_d_ of 72.6 keV/μm, at which point it achieved a measured maximum. For y_d_ sampled above 72.6 keV/μm, the H460 β-component followed the trend of the α-component and slowly decreased. A pure α-component fit for H460 was found only for the highest y_d_ tested for carbon ions, 270.3 keV/μm (Figure 3C). For H1437 cells, the β decreased with exposure to increasing y_d_, effectively reaching zero for exposures to carbon ions with y_d_ of 72.6 keV/μm and above (Figure 3D).

**Figure 4:**
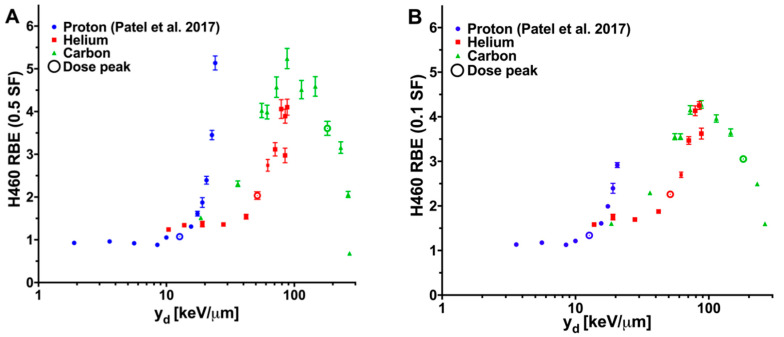

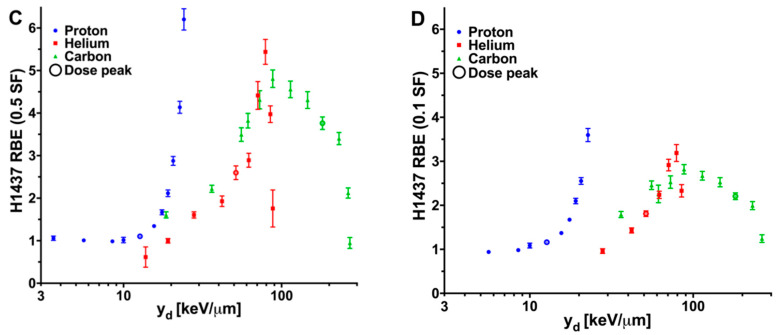
Proton, helium and carbon ion relative biological effectiveness (RBE) versus y_d_. RBEs were calculated at surviving fractions of 0.5 (RBE (0.5 SF)) and 0.1 (RBE (0.1 SF)). Irradiations at the Heidelberg Ion Therapy Center consisted of exposing cells to protons (blue), helium ions (red), and carbon ions (green). Previously published results for cells exposed to protons at the MD Anderson Proton Therapy Center are shown (purple). The location sampled closest to the Bragg peak is designated with a circle. (**A**) H460 RBE (0.5 SF). (**B**) H460 RBE (0.1 SF). (**C**) H1437 RBE (0.5 SF). (**D**) H1437 RBE (0.1 SF). The helium and carbon ion datasets demonstrated a measured increase in RBE until a y_d_ value between 79.0 and 87.9 keV/μm at which point the RBEs were found to reduce from the maximum. This feature was not present in the proton RBE. Error bars are standard error.

Helium and carbon ions produced a unique pattern of RBEs relative to RBEs we reported previously for proton irradiations [26,27]. Specifically, for H460 cells irradiated with helium, the RBE (0.5 SF) and RBE (0.1 SF) were found to increase from respective initial values of 1.24 and 1.17 for y_d_ of 10.4 keV/μm (present at the shallowest depth tested) to a maximum RBE (0.5 SF) value of 4.10 at 88.0 keV/μm and a maximum RBE (0.1 SF) value of 4.25 at 84.9 keV/μm (Figure 4). The RBE declined for y_d_ sampled above these values.

For H460 cells, the RBE for carbon ions followed a pattern similar to that of the RBE for helium ions. The entrance RBE was measured at 18.6 keV/μm, producing an SF (0.5) of 1.51 and an SF (0.1) of 1.60. The RBE (0.5 SF) value increased with y_d_ to a maximum value of 5.24 at y_d_ of 87.9 keV/μm (Figure 4A). The maximum RBE (0.1 SF) value of 4.28 was also found at 87.9 keV/μm (Figure 4B). Above this y_d_, the RBEs were found to decrease, with the lowest RBE (0.5 SF) of 0.68 present at the highest y_d_ sampled, 270.3 keV/μm. The RBE values of helium and carbon ions for H460 cells are listed in Table 1.

The RBE trends obtained for the H1437 cell line for all ion irradiations were similar to those for the H460 cells. H1437 cells irradiated with helium or carbon ions both exhibited increasing RBE values as the y_d_ increased, followed by an eventual peak response and subsequent decline. The maximum RBE (0.5 SF) for the H1437 cells was found to be 5.44 for cells exposed to 79.0 keV/μm helium ions and 4.81 for cells exposed to 87.9 keV/μm carbon ions (Figure 4C). The maximum RBE (0.1 SF) measured for the helium ion irradiations was 3.19 at 79.0 keV/μm and 2.82 at 87.9 keV/μm for the carbon ions (Figure 4D). The RBE values of helium and carbon ions for H1437 cells are listed in Table 2.

## 3. Discussion

We describe here the application of a high-throughput irradiation method to spatially map clonogenic survival of two lung cancer cell lines after exposure to particle beams. This work, coupled with our previously published results, demonstrates the reproducibility of this system between two institutions using 6 MV x-rays and protons and is the first to examine the response of H460 and H1437 cells to carbon and helium ion irradiation [26,27].

**Figure 5:**
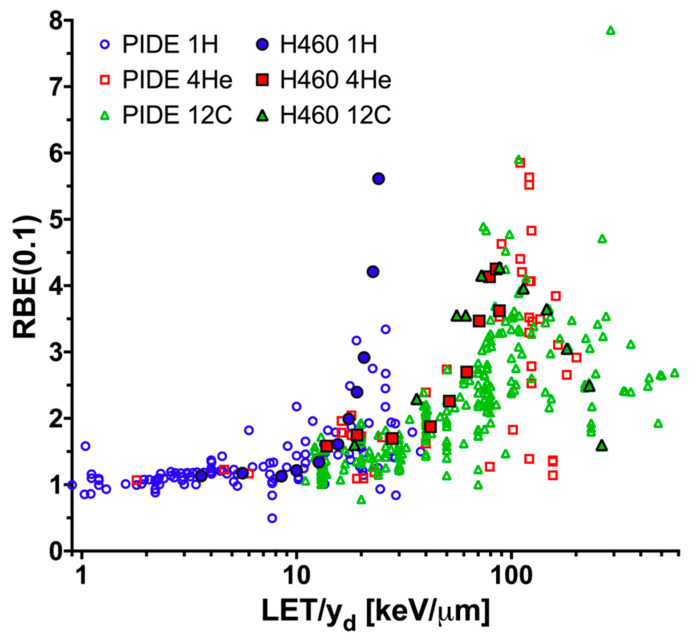
Relative biological effectiveness (RBE) versus LET/y_d_ results in the context of the PIDE database and the H460 data from the current study. The RBE was calculated at a surviving fraction level of 0.1 (RBE (0.1 SF)) for cells exposed to protons (blue circles), helium ions (red squares), or carbon ions (green triangles). The H460 results from the current study (solid shapes; black outline) and the data present in the PIDE database (open shapes) are plotted for visual comparison. LET is used for the PIDE data while y_d_ is used for the current H460 data.

It is difficult to compare the obtained results with literature RBE values, most of which were provided as a function of average LET (not indicated dose- or track-averaged). Absolute values for, and trends measured between, RBE and LET/y_d_ for proton, helium, and carbon ions for H460 and H1437 cells are similar to those reported in the literature and compiled within the Particle Irradiation Data Ensemble (PIDE) 3.2 database for other cell lines (Figure 5) [7]. For irradiation with both helium and carbon, an overkill effect, that is the decline in biological effectiveness at high y_d_ values, was noted for the highest y_d_s tested. This effect was not observed with protons. Please note that in Figure 5, LET is used for the PIDE data, while y_d_ is used for the H460 data in the current study. Because the experimental conditions and cell lines in the PIDE database were different from the current study, the data points in Figure 5 are only used for visual comparison, rather than for quantitative analysis.

Our experimental results demonstrate that even at the same LET/y_d_, the RBE of protons is higher than those of helium and carbon ions. This finding confirms that RBE is ion-specific and further indicates that using “average” physical beam characteristics cannot accurately correlate biological response among different ion species. The literature also shows that at the same LET (pure, not averaged), high-Z particles can result in a lower RBE, probably due to the escape of more delta rays from the primary particle interaction site, and the literature also recommends correlating particle track structures with biological responses [29,30].

Determining the underlying mechanisms behind the observed differences in clonogenic RBE between ours and others as a function of beam quality, such as y_d_, particularly for the higher y_d_ values tested, is a complex task. One source of variation could lie in the experimental setup. Much of the information on high-LET interactions in the articles referenced above was measured with either Van de Graff generators, cyclotrons, or radioactive sources that can produce monoenergetic charged particle beams with very narrow energy spectra even for low energies. In contrast, the work we present here consists of a monoenergetic beam produced from a synchrotron with energy modulation achieved via the traversal of the polymethyl methacrylate (PMMA) plate holder resulting in a broadened energy spectrum at the target volume. In addition to differences in the range of energies present for the primary particles, the experimental setup also contributes secondary particles to the dose and the beam quality. Some preclinical studies have specifically attempted to reduce the amount of secondary particle contamination and present results related to primary particles [31]. In addition to a larger primary particle energy spectrum at any given location, setups which use material attenuation for energy modulation result in unavoidable secondary particles, which alter the radiation fluence experienced by the biological sample. It is unlikely that the biological effect of this complex beam of radiation will be accurately described by LET, even averaged LET, as this term is defined to only describe the specified type of charged particles rather than a mixed radiation field. In contrast, to account for the radiation quality of a beam with mixed particle species present within our experimental setup we used the microdosimetric quantity lineal energy which takes into consideration the energy depositions from the total particle fluences present within the sensitive volume and has been used in several biophysical models [3,23,32,33,34,35,36,37,38]. The saturation-corrected dose-mean lineal energy (y*) has been used in the mMKM model by Kase et al. for different radiation types [34]. Their work has shown a monotonic increase trend of α derived from the linear-quadratic model with y*. However, our experimental data do not have this variation trend of α vs. y*. In addition, a constant β in the linear-quadratic model has been used in the mMKM independent of the radiation type. However, such an invariant β was not observed in our experimental results. The y* values of all ions for the present study can be found in the Tables S1–S5, calculated with the saturation parameter of y_0_ = 150 keV/µm.

An additional confounding factor is the differences in simulation methods used to calculate dose and the physical parameters of y_d_ and LET_d_ between our group and numerous others studying charged-particle RBE. The effect of the Monte Carlo (MC) simulations on the dose and beam quality calculations must be carefully considered. For any given particle, even in the simplest geometry of a homogeneous water medium, different MC packages could lead to different dose and beam quality values [39]. The simulation influences the experimental results by altering the “independent variables” of the experiments, such as the dose and beam quality (LET/y_d_). Current limitations of the knowledge of the physical description of particle interactions, especially at low energies and for materials aside from water, complicate the data derived from MC simulations, a challenge we endeavored to address in our system. Because all iterations of our high-throughput irradiation apparatus are constructed with PMMA, we have expended considerable effort to determine the physical parameters of PMMA to be used in the MC simulations. The effects of material density, mean excitation potential, tracking step size, secondary particle cut values, target volume size, and selected physics lists in Geant4 have been investigated, in part because of the need to understand these metrics for this work [27].

LET/y_d_ effects aside, an additional consideration for RBE comparisons is inherent biological sensitivity to particle radiation. Many studies examining the clinical relevance of various charged particle therapies have used either non-human or non-cancer-derived cell lines. Although the DNA damage response is a highly conserved pathway between species, mutations carried by cancer cells result in considerable variation in response. Cancer cell lines derived from human cells of the same type of cancer and irradiated under the same proton irradiation conditions have been observed with strikingly different RBEs [40].

Given the high-throughput nature of our system, characterization of differential biological responses in other tumor types is feasible and currently underway. The largest issue to consider when performing the clonogenic assay in a 96-well microplate is the reduced growth area which limits suitable cell lines to ones which form clearly distinct colonies. Circumventing this limitation will likely be achieved through the application of immunofluorescent staining techniques combined with the high-throughput system. It has recently been shown that the relative change in the number of DAPI- stained nuclei in such studies correlates well to SF [41]. This approach coupled with the high-throughput irradiation method could be used to simultaneously perform mechanistic studies and determine a pseudo-clonogenic RBE in future experiments. Aside from clonogenic RBE, of major importance, are recent studies demonstrating unique biological responses after heavy ion therapy, in particular reduced metastatic potential and host-immune activation against the tumor [42,43,44,45,46,47].

Practically, although protons certainly offer improved dose distributions over photon-based therapies, with the potential to reduce toxicity to normal tissues and late effects, the region of the beam exhibiting increased biologic activity, at and beyond the Bragg peak, is small in comparison to the total beam path. In this regard, helium or carbon ions may present a more ideal differential biological effect when integrated over clinically relevant volumes. Helium ions have recently received attention for several physical characteristics that may warrant further clinical consideration; specifically, the moderate LET of helium ions may be more sparing of normal tissues, potentially enabling the treatment of pediatric patients with better clinical outcomes. Additional benefits may be afforded from helium’s inherent stability which would reduce the off-target dose contributions seen with heavier ion therapies from their longer fragmentation tail and larger secondary particle halo [48]. Furthermore, while our initial studies have focused on the development and implementation of the high-throughput technique to measure charged particle RBE, the application of the method could be readily broadened by mapping the spatial dependence of additional forms of therapeutic energy-dependent radiation interactions such as those leveraged in boron neutron capture therapy or nanoparticle radiation enhancement [49,50,51,52,53].

## 4. Materials and Methods

### 4.1. Physical Setup and Charged Particle Irradiation Strategy

The high-throughput irradiation setup and method have been described previously [26,27]. Briefly, a 96-well microplate holder was designed out of PMMA such that a stair-step pattern with steps aligning with each column was present between the plate bottom and the beam nozzle. The increasing thickness of each step ensures that each column within the plate samples a different spatial region of the beam and receives a unique combination of dose and radiation quality. There are three ion-specific attenuation devices (jigs) according to the depth dose profiles (Figures S1–S4). All of these jigs were fabricated with a high-accuracy (±3 µm) milling machine at MD Anderson. An experimentally benchmarked Geant4 [54,55,56] MC simulation platform was used to determine the dose delivered to each column and to calculate the radiation quality summarizing parameter of dose-mean lineal energy (y_d_) in a 2-μm diameter sphere simulating a cell nucleus. LET_d_ was also calculated to enable direct comparison with our previous proton results as well as studies in the literature. Physical parameters are given in Table S6. Cells were irradiated at the Heidelberg Ion-Beam Therapy Center (HIT) in collaboration with Deutsches Krebsforschungszentrum (DKFZ) in Germany.

### 4.2. Cell Culture

H460 and H1437 lung cancer cell lines were purchased from LGC Standards GmbH (Wesel, Germany), and H460 cells were also purchased from the American Type Culture Collection (Manassas, VA, USA). Cells were cultured in a humidified incubator at 37 °C with 5% CO_2_ in RPMI 1640 media supplemented with 10% fetal bovine serum and 1% penicillin-streptomycin.

### 4.3. High-Throughput Clonogenic Assay

On the day of irradiation, cells were detached, viable cell concentration determined with a hemocytometer, and seeded at 100 cells/well. The cells were allowed to attach and normalize in culture for 8–10 h before irradiation. Plates were transferred directly from the incubator to the holder apparatus, promptly irradiated, and immediately returned to culture. Control plates were sham-irradiated. Two plates per exposure were irradiated, resulting in 16 replicates for each dose-y_d_ combination. Colonies were allowed to form for a cell line-specific amount of time (H460: 5.5 days; H1437: 7.5 days) depending on population doubling time. Cells were then stained with a 0.5% crystal violet in ethanol solution. Plates were imaged by the Texas A&M Institute of Biosciences and Technology High Throughput and Screening Center on an INCell Analyzer 6000. GE Developer software was used to score colonies consisting of at least 50 cells.

### 4.4. Surviving Fraction Analysis and RBE Calculation

Surviving fractions (SFs) were determined by normalizing the number of scored colonies in each well by the pooled plating efficiency (PE) determined from the unirradiated plates. The SF replicates were then averaged to determine the overall SF for each dose-y_d_ combination and ion species. The limit of detection below which the data was excluded was defined as the inverse of the experimental PE which resulted in lower SF limits of 0.02 for H460 and 0.03 for H1437. Colony count data were fit to the LQ cell survival model as a function of dose using Poisson regression. The RBEs were calculated from the data fits at SFs of 0.5 (RBE (0.5 SF)) and 0.1 (RBE (0.1 SF)) by comparison to previously generated Cs-137 photon datasets [26].

### 4.5. Statistical Analyses

Plotting, fitting, and statistical testing were done with GraphPad Prism 8.0. LQ model fits were achieved by Poisson regression to the clonogenic data. LQ model fits and standard errors were confirmed using the CFAssay analysis package in R [57]. The extra sum-of-squares *F* test was used to determine if model fits to clonogenic data were significantly different (*p* < 0.05). RBE uncertainties were calculated by propagating the uncertainties and covariances associated with the LQ model fit parameters.

## 5. Conclusions

Compared with traditional experimental methods, high-accuracy and high-throughput methods such as the one presented here, are essential to advance the development of biophysical models to the unique conditions present within the therapeutic region of the beam as well as the fragmentation tail of heavier ion beams. Likewise, further study is required to map the range of biological outcomes for the numerous subtypes of cancer. The results presented here show that the application of the high-throughput biophysical system developed can improve the efficiency in producing biological response data to aid in this pursuit. Ultimately, standardized biology techniques, designed to associate physical factors with biological response, have the potential to characterize individual charged particle beams. The clonogenic cell survival data and the corresponding microdosimetric data obtained from the present work can be used to further validate the existing microdosimetry-based RBE models such as the stochastic microdosimetric kinetic model (SMK) [3], and the repair–misrepair–fixation (RMF) model [36].

## Figures and Tables

**Table 1:** Heavy ion RBE values for H460 cells.

Column	Helium Ions			Carbon Ions		
	y_d_, keV/µm	RBE (0.5 SF) *	RBE (0.1 SF) *	y_d_, keV/µm	RBE (0.5 SF) *	RBE (0.1 SF) *
1	10.4	1.24	**	18.6	1.51	1.60
2	13.8	1.34	1.58	36.2	2.31	2.29
3	19.1	1.37	1.75	55.6	4.02	3.55
4	27.9	1.36	1.70	61.3	3.99	3.55
5	42.0	1.54	1.88	72.6	4.57	4.15
6	51.4	2.03	2.26	87.9	5.24	4.28
7	62.0	2.74	2.70	113.8	4.51	3.96
8	70.8	3.12	3.47	146.0	4.59	3.65
9	79.0	4.06	4.13	181.3	3.61	3.05
10	84.9	3.89	4.25	230.8	3.16	2.49
11	88.0	4.10	3.62	263.6	2.06	1.60
12	84.7	2.97	**	270.3	0.68	**

* refers to RBE at surviving fractions of 0.5 or 0.1. ** value excluded due to lack of data coverage. Abbreviations: y_d_, dose-mean lineal energy; RBE, relative biological effectiveness; SF, surviving fraction.

**Table 2:** Heavy ion RBE values for H1437 cells.

Column	Helium Ions			Carbon Ions		
	y_d_, keV/µm	RBE (0.5 SF) *	RBE (0.1 SF) *	y_d_, keV/µm	RBE (0.5 SF) *	RBE (0.1 SF) *
1	10.4	**	**	18.6	1.61	**
2	13.8	0.61	**	36.2	2.22	1.79
3	19.1	1.00	**	55.6	3.50	2.46
4	27.9	1.61	0.96	61.3	3.82	2.26
5	42.0	1.93	1.43	72.6	4.31	2.53
6	51.4	2.60	1.81	87.9	4.81	2.82
7	62.0	2.89	2.24	113.8	4.56	2.67
8	70.8	4.41	2.91	146.0	4.31	2.52
9	79.0	5.44	3.19	181.3	3.76	2.21
10	84.9	3.97	2.33	230.8	3.40	1.99
11	88.0	1.76	**	263.6	2.12	1.24
12	84.7	**	**	270.3	0.95	**

* refers to RBE at surviving fractions of 0.5 or 0.1. ** value excluded due to lack of data coverage. Abbreviations: y_d_, dose-mean lineal energy; RBE, relative biological effectiveness; SF, surviving fraction.

## Supplementary Materials

The following are available online at https://www.mdpi.com/2072-6694/12/12/3658/s1, Figure S1: The experimental setup, Figure S2: The depth dose profile of experimental protons at HIT, Figure S3: The depth dose profile of experimental helium ions at HIT, Figure S4: The depth dose profile of experimental carbon ions at HIT, Table S1: H1437 linear-quadratic fitting parameters from protons at HIT, Table S2: H460 linear-quadratic fitting parameters from helium ions at HIT, Table S3: H1437 linear-quadratic fitting parameters from helium ions at HIT, Table S4: H460 linear-quadratic fitting parameters from helium ions at HIT, Table S5: H1437 linear-quadratic fitting parameters from carbon ions at HIT, and Table S6: Physical parameters of the helium and carbon ion irradiation setups.
