# Supplementary material for: Mapping the Relative Biological Effectiveness of Proton, Helium and Carbon Ions with High-Throughput Techniques
Source: Cancers (Basel). 2020 Dec 5;12(12):3658. doi: 10.3390/cancers12123658 (PMC7762185; doi:10.3390/cancers12123658)
Supplement: Supplementary file 1 [file cancers-12-03658-s001.pdf]

Mapping the Relative Biological Effectiveness of Proton, Helium and Carbon Ions with High-Throughput Techniques

Lawrence Bronk, Fada Guan, Darshana Patel, Duo Ma, Benjamin Kroger, Xiaochun Wang, Kevin Tran, Joycelyn Yiu, Clifford Stephan, Jürgen Debus, Amir Abdollahi, Oliver Jäkel, Radhe Mohan, Uwe Titt and David R. Grosshans

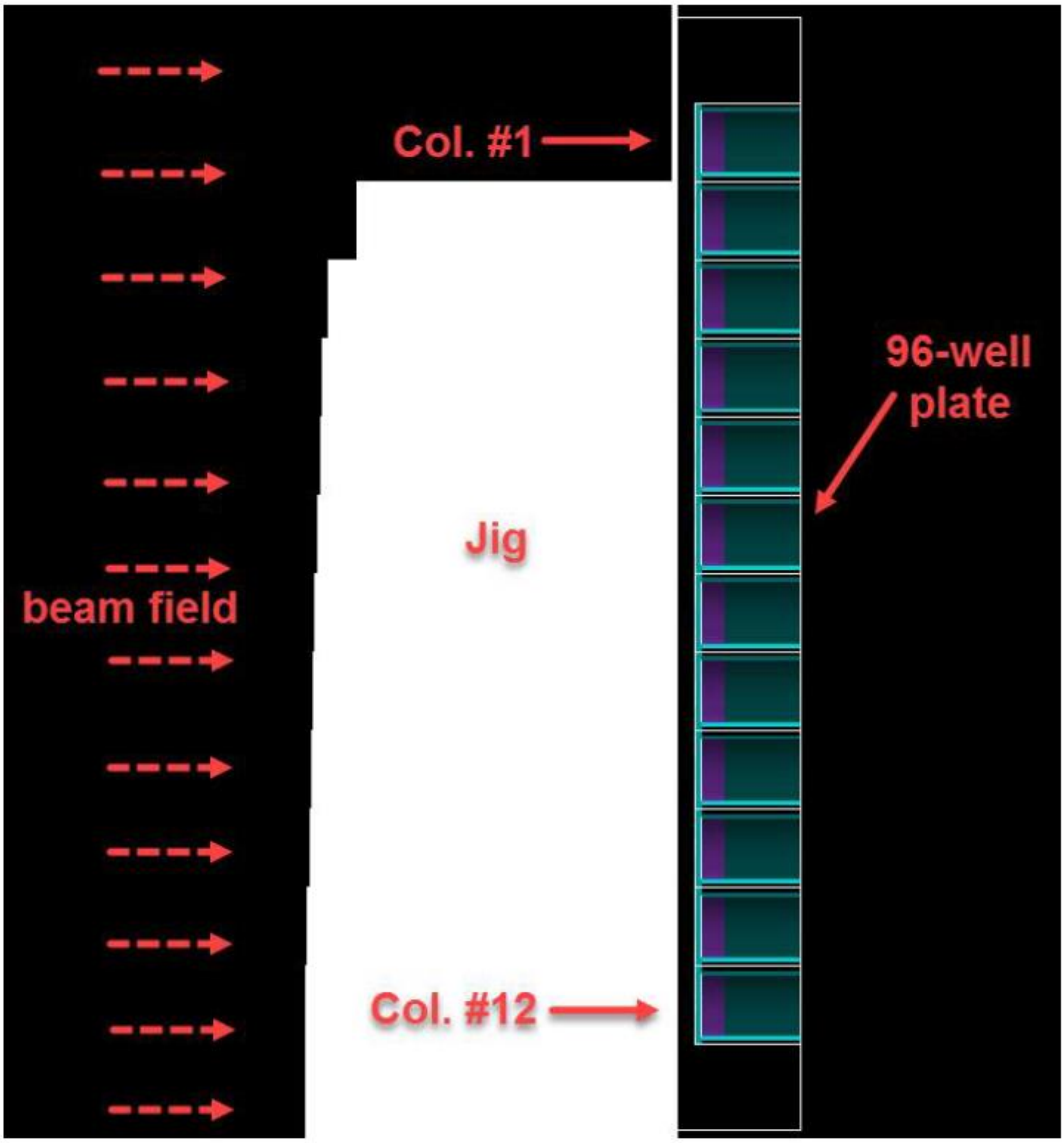

Figure S1. The experimental setup (modeled using Geant4) at HIT with a horizontal beam irradiating the jig (made of PMMA) and a 96-well cell culture plate with cells attached to the bottom of each well. Proton jig is illustrated here as an example. For each ion species, there is a specific jig designed according to its depth dose profile and the sampled locations. The thickness of each step was determined according to the spatial location selected from the depth dose curve.

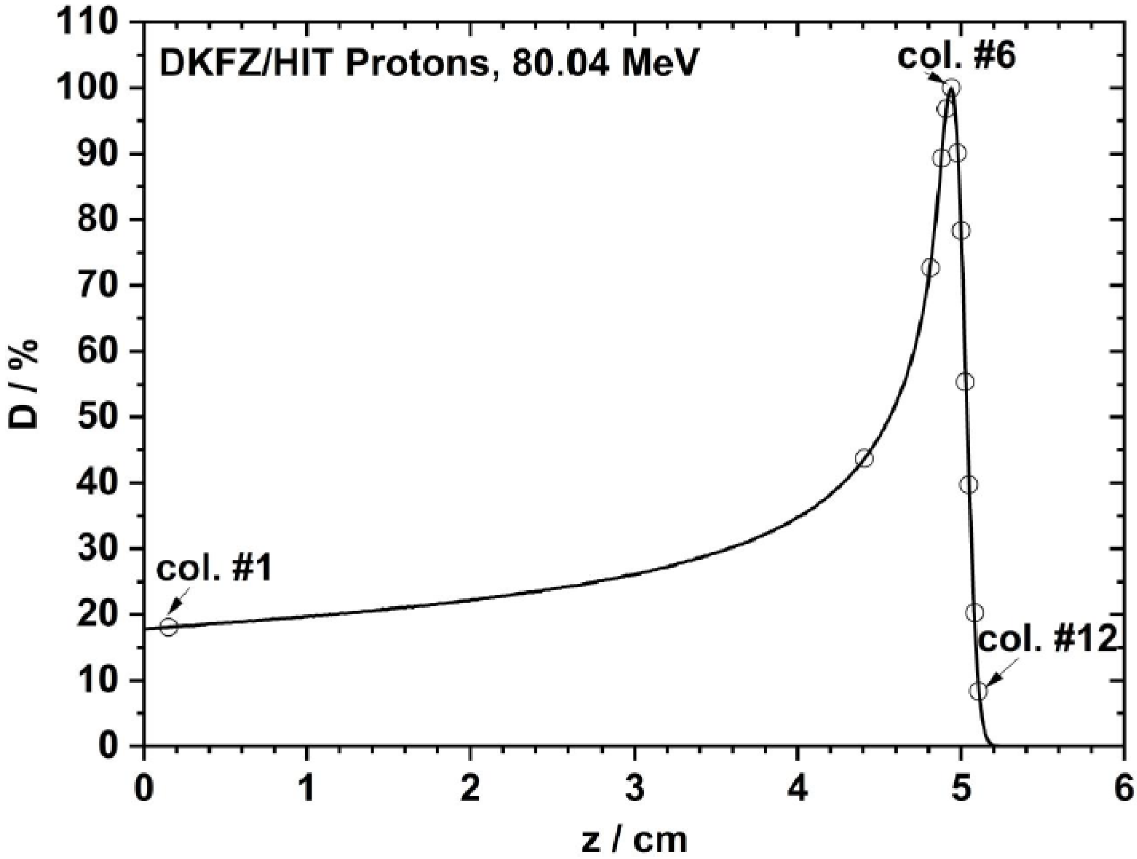

Figure S2. The depth dose profile (in water) of the proton beam at HIT with the nominal energy of 80.04 MeV. The 12 locations used to sample the biological effect are marked with the circle.

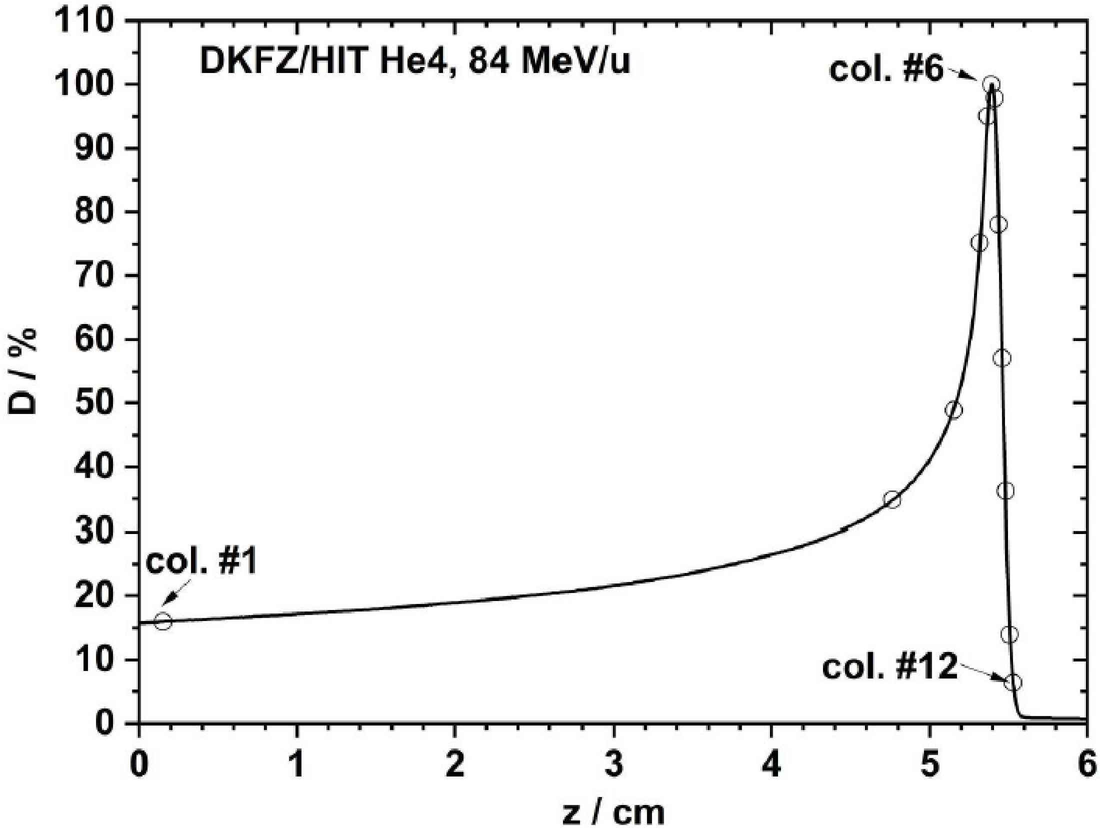

Figure S3. The depth dose profile (in water) of the Helium-4 ion beam at HIT with the nominal energy of 84 MeV/u. The 12 locations used to sample the biological effect are marked with the circle.

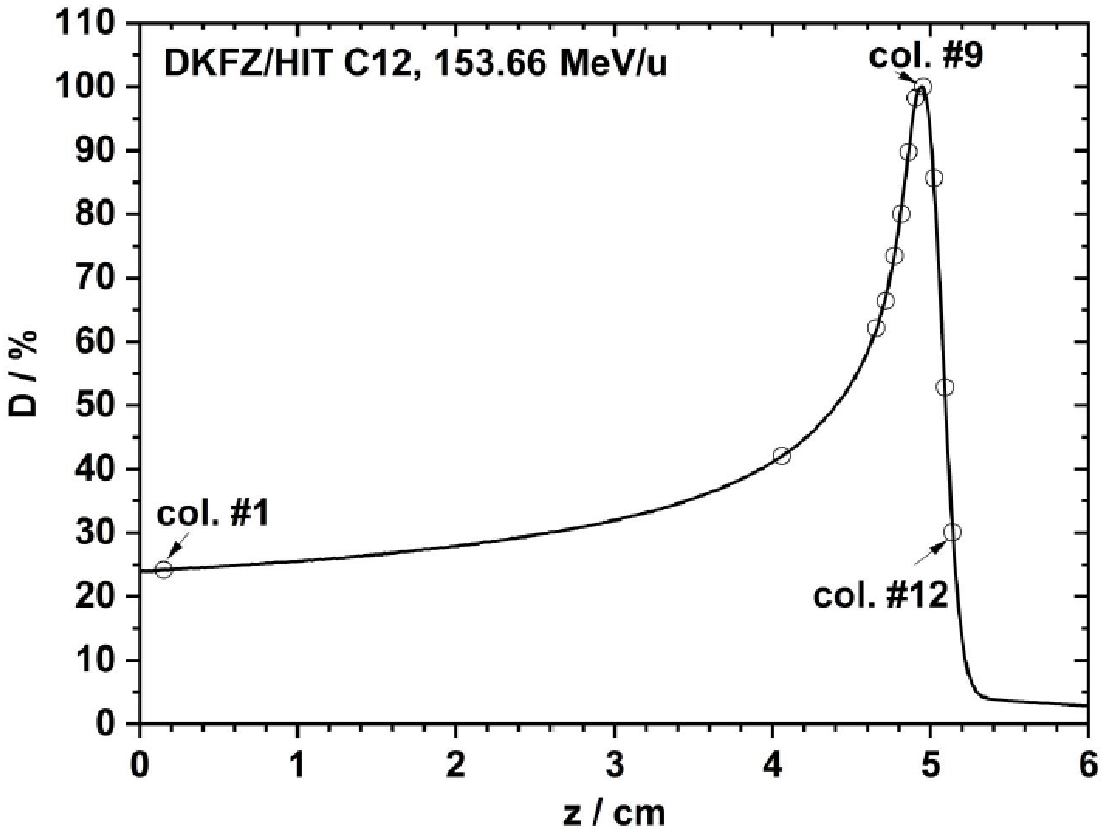

Figure S4. The depth dose profile (in water) of the Carbon-12 ion beam at HIT with the nominal energy of 153.66 MeV/u. The 12 locations used to sample the biological effect are marked with the circle.

Table S1. H1437 linear-quadratic fitting parameters from proton irradiations done at the Heidelberger Ionenstrahl Therapiezentrum.

Column	LETd [keV/μm]	yd [keV/μm]	y* [keV/μm]	α	β	αSE	βSE	RBE (0.5) *	RBE (0.1) *
1	1.0	1.9	1.8	0.1167		0.0692		**	**
2	2.6	3.6	3.5	0.1432	0.0187	0.0452	0.0118	1.06	**
3	4.7	5.6	5.5	0.0897	0.0302	0.0299	0.0055	1.01	0.94
4	7.3	8.5	8.3	0.0508	0.0390	0.0271	0.0047	0.99	0.98
5	8.7	10.0	9.8		0.0565		0.0051	1.02	1.09
6	11.1	12.7	12.4	0.0140	0.0622	0.0372	0.0076	1.10	1.16
7	13.7	15.6	15.0	0.0490	0.0802	0.0501	0.0117	1.34	1.37
8	15.4	17.5	16.8	0.0791	0.1150	0.0616	0.0173	1.67	1.67
9	16.9	19.1	18.3	0.1166	0.1754	0.0840	0.0304	2.12	2.10
10	18.3	20.6	19.7	0.3191	0.1945	0.0832	0.0374	2.88	2.55
11	20.2	22.7	21.6	0.4913	0.3649	0.1195	0.0867	4.14	3.60
12	21.4	24.1	22.9	0.5370	1.1680	0.2765	0.3740	**	**

*refers to RBE at surviving fractions of 0.5 or 0.1 with the reference radiation of Cs-137 photons. **value excluded due to lack of data coverage. Abbreviations: LETd, dose-averaged linear energy transfer; y_d, dose-mean lineal energy; y*, saturation-corrected dose-mean lineal energy (calculated with the saturation parameter of y_0 = 150 keV/μm); SE, standard error from the data fitting; RBE, relative biological effectiveness.

Table S2. H460 linear-quadratic fitting parameters from helium ion irradiations done at the Heidelberger Ionenstrahl Therapiezentrum.

Column	yd [keV/μm]	y*	α	β	α SE	β SE

		**[keV/µm]**				
1	10.4	4.8	0.4059	0.0936	0.0657	0.0359
2	13.8	10.6	0.1726	0.3283	0.0587	0.0296
3	19.1	16.1		0.4894		0.0323
4	27.9	25.0	0.0611	0.4325	0.0617	0.0299
5	42.0	35.3	0.1304	0.4982	0.1066	0.0646
6	51.4	41.1	0.4082	0.5738	0.1212	0.0835
7	62.0	47.1	0.8252	0.5782	0.1344	0.1003
8	70.8	51.8	0.6178	1.3590	0.2033	0.2062
9	79.0	55.9	1.1240	1.5090	0.2480	0.2990
10	84.9	58.4	0.8437	1.9470	0.2202	0.2542
11	88.0	59.1	1.4730	0.6924	0.2080	0.2298
12	84.7	55.3	1.2670		0.0977	

Abbreviation: y_d, dose-mean lineal energy; y*, saturation-corrected dose-mean lineal energy (calculated with the saturation parameter of y_0 = 150 keV/µm); SE, standard error from the data fitting.

Supplementary Table S3. H1437 linear-quadratic fitting parameters from helium ion irradiations done at the Heidelberger Ionenstrahl Therapiezentrum.

**Column**	**yd [keV/µm]**	**y* [keV/µm]**	**α**	**β**	**α SE**	**B SE**
1	10.4	4.8				
2	13.8	10.6	0.0941	0.0044	0.0492	0.0145
3	19.1	16.1	0.1791	0.0042	0.0379	0.0084
4	27.9	25.0	0.3099	0.0013	0.0310	0.0053
5	42.0	35.3	0.3144	0.0331	0.0491	0.0108
6	51.4	41.1	0.4526	0.0387	0.0637	0.0168
7	62.0	47.1	0.4439	0.0967	0.0870	0.0309
8	70.8	51.8	0.8039	0.0689	0.1088	0.0473
9	79.0	55.9	1.0590		0.0612	
10	84.9	58.4	0.7735		0.0396	
11	88.0	59.1	0.3422		0.0698	
12	84.7	55.3		0.0977		0.1649

Abbreviation: y_d, dose-mean lineal energy; y*, saturation-corrected dose-mean lineal energy (calculated with the saturation parameter of y_0 = 150 keV/µm); SE, standard error from the data fitting.

Table S4. H460 linear-quadratic fitting parameters from carbon ion irradiations done at the Heidelberger Ionenstrahl Therapiezentrum.

**Column**	**yd [keV/µm]**	**y* [keV/µm]**	**α**	**β**	**α SE**	**β SE**
1	18.6	14.3	0.3717	0.2543	0.0678	0.0348
2	36.2	31.6	0.6830	0.4294	0.0774	0.0545
3	55.6	47.2	1.4460	0.6654	0.1559	0.1487
4	61.3	50.5	1.4160	0.6943	0.1462	0.1346
5	72.6	55.2	1.5820	1.0270	0.2218	0.2397
6	87.9	59.1	2.0360	0.6316	0.1728	0.1759
7	113.8	63.4	1.6330	0.8050	0.1770	0.1793
8	146.0	66.8	1.8210	0.3770	0.1601	0.1348
9	181.3	68.8	1.3560	0.4001	0.1312	0.0995
10	230.8	68.9	1.2580	0.1679	0.1035	0.0629
11	263.6	66.2	0.8320	0.0584	0.0588	0.0252
12	270.3	62.1	0.2907		0.0225	

Abbreviation: y_d, dose-mean lineal energy; y*, saturation-corrected dose-mean lineal energy (calculated with the saturation parameter of y_0 = 150 keV/µm); SE, standard error from the data fitting.

Table S5. H1437 linear-quadratic fitting parameters from carbon ion irradiations done at the Heidelberger Ionenstrahl Therapiezentrum.

**Column**	**yd [keV/μm]**	**y* [keV/μm]**	**α**	**β**	**α SE**	**β SE**
1	18.6	14.3	0.0283	0.1284	0.0672	0.0313
2	36.2	31.6	0.3175	0.0717	0.0494	0.0168
3	55.6	47.2	0.6045	0.0748	0.0627	0.0253
4	61.3	50.5	0.7415	0.0027	0.0770	0.0386
5	72.6	55.2	0.8395		0.0368	
6	87.9	59.1	0.9360		0.0316	
7	113.8	63.4	0.8872		0.0296	
8	146.0	66.8	0.8383		0.0297	
9	181.3	68.8	0.7325		0.0247	
10	230.8	68.9	0.6621		0.0267	
11	263.6	66.2	0.4131		0.0252	
12	270.3	62.1	0.1030	0.0216	0.0529	0.0181

Abbreviation: y_d, dose-mean lineal energy; y*, saturation-corrected dose-mean lineal energy (calculated with the saturation parameter of y_0 = 150 keV/μm); SE, standard error from the data fitting.

Table S6. Physical parameters of the helium and carbon ion irradiation setups. Each column of a standard 96-well microplate was placed such that the incident beam traveled through a different PMMA thickness before impinging on the bottom of the plate. Monte Carlo simulations using the Geant4 toolkit were used to determine the LETd (primary particles only) and yd and dose within each column.

**Column**	**1**	**2**	**3**	**4**	**5**	**6**	**7**	**8**	**9**	**10**	**11**	**12**
						Helium						
PMMA thickness, mm	0	39.21	42.51	43.91	44.32	44.51	44.72	44.91	45.12	45.31	45.51	45.71
LETd, keV/μm	6.6	11.1	14.3	21.9	33.8	41.6	50.4	57.9	65.2	70.3	73.4	70.1
yd, keV/μm	10.4	13.8	19.1	27.9	42.0	51.4	62.0	70.8	79.0	84.9	88.0	84.7
Dose, rel. units	1.00	2.09	2.97	4.55	5.72	6.05	5.77	4.79	3.21	1.85	0.85	0.34
						Carbon						
PMMA thickness, mm	0	33.80	38.98	39.48	39.99	40.35	40.75	41.14	41.55	42.15	42.75	43.15
LETd, keV/μm	20.2	39.8	63.1	70.6	84.3	100.8	126.3	157.6	196.4	242.9	285.8	308.4
yd, keV/μm	18.6	36.2	55.6	61.3	72.6	87.9	113.8	146.0	181.3	230.8	263.6	270.3
Dose, rel. units	1.00	1.64	2.39	2.60	2.89	3.18	3.58	3.94	4.09	3.53	2.20	1.33

Abbreviations: PMMA, polymethyl methacrylate; LETd, dose-averaged linear energy transfer; yd, dose-mean lineal energy; rel., relative.

Publisher's Note: MDPI stays neutral with regard to jurisdictional claims in published maps and institutional affiliations.

© 2020 by the authors. Licensee MDPI, Basel, Switzerland. This article is an open access article distributed under the terms and conditions of the Creative Commons Attribution (CC BY) license (http://creativecommons.org/licenses/by/4.0/).
